# A review of diffusion-weighted magnetic resonance imaging in head and neck cancer patients for treatment evaluation and prediction of radiation-induced xerostomia

**DOI:** 10.1186/s13014-022-02178-0

**Published:** 2023-01-30

**Authors:** Tai Ermongkonchai, Richard Khor, Morikatsu Wada, Eddie Lau, Daniel Tao Xing, Sweet Ping Ng

**Affiliations:** 1 Department of Radiation Oncology, Olivia-Newton John Cancer and Wellness Centre, Austin Health, 145 Studley Road, Heidelberg, Melbourne, VIC 3084 Australia; 2 Department of Radiology, Austin Health, Heidelberg, Melbourne, Australia; 3 Department of Molecular Imaging and Therapy, Austin Health, Heidelberg, Melbourne, Australia; 4 Department of Radiology, The University of Melbourne, Parkville, Melbourne, Australia

**Keywords:** Diffusion magnetic resonance imaging, Head and neck cancer, Magnetic resonance imaging, Radiotherapy, Xerostomia

## Abstract

The incidence of head and neck cancers (HNC) is rising worldwide especially with HPV-related oropharynx squamous cell carcinoma. The standard of care for the majority of patients with locally advanced pharyngeal disease is curative-intent radiotherapy (RT) with or without concurrent chemotherapy. RT-related toxicities remain a concern due to the close proximity of critical structures to the tumour, with xerostomia inflicting the most quality-of-life burden. Thus, there is a paradigm shift towards research exploring the use of imaging biomarkers in predicting treatment outcomes. Diffusion-weighted imaging (DWI) is a functional MRI feature of interest, as it quantifies cellular changes through computation of apparent diffusion coefficient (ADC) values. DWI has been used in differentiating HNC lesions from benign tissues, and ADC analyses can be done to evaluate tumour responses to RT. It is also useful in healthy tissues to identify the heterogeneity and physiological changes of salivary glands to better understand the inter-individual differences in xerostomia severity. Additionally, DWI is utilised in irradiated salivary glands to produce ADC changes that correlate to clinical xerostomia. The implementation of DWI into multi-modal imaging can help form prognostic models that identify patients at risk of severe xerostomia, and thus guide timely interventions to mitigate these toxicities.

## Introduction

Head and neck cancer (HNC) of the mucosa is increasing worldwide, currently accounting for up to 4% of all malignancies [1]. More than 60% of presentations are locally advanced tumours, with most fatalities caused by uncontrolled locoregional disease [2, 3]. Approximately 90% of HNCs are of squamous cell carcinoma (SCC) type, with alcohol and smoking being the significant risk factors [4]. However, in the past decade, there has been a decline in the rates of HNC from those with ‘traditional’ risk factors of smoking and alcohol, and a rise in those with human papillomavirus (HPV)-related oropharyngeal SCC who are typically young, non-smokers. Patients with human papillomavirus (HPV)-related oropharyngeal SCC have better survival outcomes with treatment [3, 5]. Curative-intent radiotherapy (RT) is the standard of care for patients with pharyngeal (nasopharynx, oropharynx, and larynx) carcinomas as modern technological advancements, altered fractionation schemes and the addition of concurrent chemotherapy have achieved excellent efficacy in locoregional control (LRC) and long-term survival. With improving outcomes, survivorship now focuses on management of RT-related late toxicities [6]. Toxicities are often classified into acute (onset from day 1–90) and late (onset from day 91 to the remainder of life) [7]. Xerostomia is a late toxicity of concern as it constitutes a significant quality-of-life (QoL) burden, and is often unavoidable due to the involvement of salivary glands in therapy volumes [8]. This has changed the way normal structures are accounted for during treatment planning. There has been a progressive shift in survivorship research towards incorporating magnetic-resonance imaging (MRI) and its functional imaging features to develop prognostic models that will predict late toxicities. Diffusion-weighted imaging (DWI) is an MRI function that is of recent interest due to its accurate evaluation of changes in cellular architecture [9]. This review aims to address the uses of DWI in evaluating HNC treatment outcomes and its emerging potential in predicting radiation-induced toxicities.

For this review, a comprehensive search was conducted on PubMed during April to May 2021, using Medical Subject Headings (MeSH) of head and neck cancer, radiotherapy, magnetic resonance, and diffusion magnetic resonance imaging. Studies were screened using specific selection criteria, and were included in this review if they reported use of DWI/MR imaging for evaluation of salivary glands and/or treatment assessment of HNCs. Literature were excluded if they studied oligometastatic disease, previously resected tumours or explored novel adjuvant therapies. Only results in the English language were included.

### Chemoradiotherapy in head and neck cancer

Traditionally, conventional RT such as computed tomography (CT)-based three-dimensional conformal radiotherapy (3DCRT) have had limitations on protecting normal tissues, resulting in considerable toxicity rates and modest disease locoregional control (LRC) [10, 11]. This led to the introduction of Intensity Modulated Radiotherapy (IMRT), a technique that enables organs at risk (OAR) sparing by ensuring a steep dose gradient between the tumour target and normal structures [12, 13]. The PARSPORT trial demonstrated this by sparing parotid glands, thus lowering rates of radiation-induced xerostomia, improving salivary function recovery and achieving greater QoL compared to conventional techniques [14]. Furthermore, subsequent studies proposed the addition of concurrent chemotherapy to RT plans and confirmed its benefit towards treatment outcomes [15]. Platinum-based chemotherapy such as cisplatin became the agent of choice [16–18]. A trial by Chitapanarux et al. compared treatment outcomes of a concurrent chemo-radiotherapy (CCRT) arm to an accelerated RT arm [19]. The CCRT group achieved better LRC and statistically improved overall survival (OS) compared to the accelerated arm (p = 0.05) [19]. Ghadjar et al. also showed similar benefits with CCRT in terms of LRC and OS [20], while Gupta et al. reported comparable local tumour control rates between CCRT and accelerated RT [17]. As a result, concurrent chemo-radiotherapy with IMRT has emerged as the standard of care for unresectable HNC. However, despite the improvement in treatment outcomes, CCRT still accrues considerable toxicity because the combination of RT and chemotherapy inflicts more structural damage [20]. This review will focus on RT-related xerostomia, since it contributes to a significant QoL burden and is under-recognised and under-estimated clinically [21].

### Xerostomia

Radiation-related xerostomia is dry mouth resulting from radiation-induced damage to the salivary glands. It is a subjective assessment that is difficult to quantify due to discrepancies between clinician and patient perceptions [21]. The parotid and submandibular glands are the major tissues responsible for salivary output; parotid glands contribute up to 70% of salivary flow during gustatory stimulation, while submandibular glands supply up to 90% of salivary output during rest [22]. Parotid glands are highly radio-sensitive [23] and has been shown to undergo glandular atrophy and morphological changes as soon as during the early stages of an RT course [8]. Furthermore, it is difficult to spare these critical glands during treatment because they are close to or within the Planning Target Volume (PTV), hence sparing these structures often compromises tumour control [23]. This explains why radiation-induced xerostomia is the most frequently reported late toxicity amongst HNC patients [24], with subjective xerostomia reporting having strong correlations with mean RT dose to the oral cavity [21].

Additionally, the development of xerostomia is not just determined by mean total dose. Buettner et al. demonstrated that the spatial distribution of doses between the superficial and deep lobes of the parotid glands also impact toxicity [23]. This implies that the parotid glands contain regions of varying radio-sensitivities. A study by Nevens et al. found that there is no significant difference between sparing both parotid glands and sparing just the contralateral gland, however, sparing one whole gland leads to better late xerostomia outcomes compared to sparing just one superficial lobe [25]. One hypothesis to explain this phenomenon is the distribution of salivary gland stem cells. These drive the renewal of parotid gland tissue after radiation damage and are dispersed in regions throughout the glands [25]. Since the parotids exhibit varying radio-sensitivities and the rates of patient recovery from late xerostomia differ, it can be assumed that heterogeneity exists between each individual in the distribution of parotid stem cells. It is also postulated that parotid glands contain functional regions arranged in parallel, whereby xerostomia occurs when there is destruction of a sufficient number of glandular units or significant damage to key functional regions [26]. Therefore, if a treatment plan aims to optimise parotid gland sparing, it must consider the dosage distribution in addition to mean total dose.

### Adaptive radiotherapy and MRI imaging

Adaptive radiotherapy (ART) is an approach that aims to address the complexities of intra-treatment glandular changes. It incorporates imaging during the treatment course, so that RT plans can be re-adapted according to anatomical movements and the shrinking tumour volume [2, 4]. A dosimetric study by Kataria et al. showed that the use of ART reduced the volume of parotid glands receiving significant radiation dose [2]. Meanwhile, Maheshwari et al. suggests that ART re-scanning also achieves improvement in local tumour control as it provides insight into tumour volume reduction [4]. Early ART methods use CT imaging [2], however Magnetic-Resonance Imaging (MRI) also offers additional benefits. Firstly, MRI’s soft-tissue contrast and image resolution is superior to CT [27–29], which would enable more accurate delineation of tissue changes. High resolution MRI can demonstrate intricate morphological information of the parotid glands, therefore visualising the radiation-induced changes that occur to the duct system and connective tissue septa [8]. Moreover, MRI does not expose patients to additional radiation, unlike CT [27]. This enables continuous MRI scans to be taken repeatedly throughout treatment and acquire real-time images that depict subtle intra-treatment changes. Its lower safety risks relative to CT also makes it suitable for longitudinal studies with lengthy follow-up times.

The advantages of MRI’s have also been deployed in other aspects of HNC management. Bruijnen et al. utilised cine MR imaging to quantify respiratory- and swallowing-induced intra-fractional tumour motion for nasopharyngeal, oropharyngeal and laryngeal cancers, such that adequate PTV margins (or internal target volume, ITV) can be applied for each HNC subtype [30]. It is paramount that PTV’s are appropriate because underestimation can lead to missing the geometric target tumour, while overestimation will irradiate more of the surrounding OARs. Meanwhile, other research have explored the use of MRI signal changes to predict clinical toxicity. Van Dijk et al. used MRI imaging biomarkers to estimate fat concentration of parotid glands, where increased lipid density correlated to higher risks of late xerostomia [31]. Hence, instead of aiming to eliminate toxicities—an unattainable task—research has shifted towards optimising imaging modalities to identify at-risk cohorts.

Furthermore, MRI’s excellent imaging quality has made it easier for artificial intelligence (AI) and deep-learning machines to be integrated into treatment planning. Doshi et al. developed an MRI-based software that automatically contours tumour volumes with reproducibility [32]. Such tools can improve workflow efficiency by reducing the need for clinicians to manually contour each structure, while also reducing inter- and intra-observer variability [32].

As the implementation of MRI with ART is still in its early stages, some caveats have emerged that poses additional challenges. Namely, this technology requires the use of the MRI magnet outside radiology departments, where such technologies would normally be subject to strict protocols. There will be an initial learning curve in establishing protocols that meet safety requirements. Concomitantly, adaptive techniques can be a resource-intensive practice as it requires increased time investment by multidisciplinary teams to review and re-adapt plans between each fraction.

### Diffusion-weighted imaging

Diffusion-Weighted Imaging (DWI) is a function of MRI being explored in HNC management. It is a non-invasive technique that measures the random motion of water molecules within different tissue compartments [1, 9, 33, 34]. Molecular motion is determined by its size, environmental temperature and the cellularity of its surroundings, therefore DWI can provide quantitative information of the diffusivity of each structure based on differences in water mobility [1, 9]. Using DWI, the user can identify and characterise different tissues as each structure possesses a unique cellular architecture profile. An important parameter of DWI is the b value, which represents the sensitivity of a DWI sequence to the effects of diffusion; higher b values equate to greater weighting of diffusion. And to quantify cellular diffusion, DWI sequences of varying b values must be generated. This allows the calculation of the Apparent Diffusion Coefficient (ADC) map [33]. ADCs are essentially values that represent the signal attenuation that occurs with molecular diffusion [9]. Thus, the hypercellular environment of a tumour impedes the motion of water molecules, appearing as increased signals on the DWI sequence and represented by low ADC values (mm^2^/s) [18, 33, 35]. Meanwhile, hypocellular environments such as necrotic tissue will exhibit high cellular diffusion characterised by high ADC [9]. This phenomenon has led to the speculation that changes in ADC values can be detected in the early stages of RT treatment, since normal tissues begin the apoptotic process from the onset of radiation exposure [36]. Larger ADC changes may represent more severe cellular damage and possibly be indicative of late toxicity. Therefore, DWI provides synergistic functional information to the MRI, as standard MRI sequences visualise the fine anatomical details of structures, whereas the ADC maps quantify the diffusivity between different tissues. Such detail is crucial to navigate the complex anatomy of the head and neck region.

Studies have begun utilising DWI as part of HNC treatment planning. Felice et al. proposes that DWI should be incorporated during tumour contouring as it can possibly delineate the target volume with more accuracy [37]. During treatment planning, Gross Tumour Volume (GTV) represents the area with confirmed disease, and the study found that the mean GTVs determined using ADC were significantly smaller than GTVs from standard CT (p = 0.0078), resulting in a reduction in dosage in all HNC cases [37]. This is highly advantageous for dose-sparing of sensitive tissues, but the increased risk of local failure from reduced target volumes should also be considered. Cardoso et al. also supports the contouring benefits of DWI, but their results were contradictory. Their study reported that GTVs delineated with DWI were larger than those done on CT alone and CT/PET (P < 0.05) [34]. The conflicting results of the two studies could be due to the heterogeneity of tumours between the patient cohorts and differing b values used. However, both reports agree that the superior visualisation and histological representation of DWI/MRI helps reduce clinician uncertainty by making the tumour target more identifiable compared to standard CT, and can lead to more accurate delineation of GTV volumes—higher or lower. Plus, the addition of DWI into treatment workup is not inconvenient as it is inexpensive, quick (acquisition takes a few minutes) and does not involve intravenous contrast [34].

### Prognostic role of diffusion-weighted imaging in head and neck tumours

A key strength of DWI is its signals not being influenced by post-treatment effects such as inflammation. This is not the case for Fluorodeoxyglucose (FDG)-Positron Emission Tomography (PET)—another popular functional imaging modality – as it has the propensity to give false-positive signals due to FDG’s avidity towards inflammatory activity [38]. Hence, DWI’s features make it a promising prognostic tool in predicting treatment outcomes for HNCs, especially during intra- or post-treatment periods where treatment-induced inflammation is ubiquitous. For instance, ADC analyses have been conducted at pre, mid- and post-treatment stages for various uses. A study by Alamolhoda et al. and a systematic review by Driessen et al. reported that the mean ADC values of benign lymph nodes were higher than of malignant nodes [1, 38], indicating a diagnostic use of DWI in detecting metastatic disease during pre-treatment workup. Meanwhile, Matoba et al. demonstrated that mid-treatment ADC changes of the primary tumour were significantly lower in patients with locoregional failure compared to those with locoregional control (p < 0.05) [36], suggesting a role for DWI to predict treatment response. A systematic review by Chung et al. showed similar reports where high ADCs during pre-treatment and smaller changes in ADCs during mid- and post-treatment phases of concurrent chemoradiotherapy indicated locoregional failure [39]. Lastly, post-treatment ADC mapping can be used to differentiate a recurrent tumour from typical post-RT tissue changes. Two studies by Abdel Razek et al. found a significant difference between the ADCs of recurrent HNC lesions (low ADCs) and post-treatment changes (higher ADCs) (p < 0.05) [40, 41], with the detection of tumour recurrence achieving a sensitivity of 94% and specificity of 100% [40].

### Diffusion-weighted imaging in normal salivary glands

The assessment of normal physiology is crucial in understanding the pathological processes of radiation-induced tissue damage. Previously, there has been two practical methods in objectively measuring salivary gland function: salivary gland scintigraphy (SGS) with technetium Tc99m pertechnetate, and manual measurement of salivary flow. DWI has been added as a non-invasive and convenient method in visualising gland function, as ADC changes occur during gustatory stimulation of salivary glands. Thoeny et al. demonstrated significant decreases in ADC values of the salivary glands within minutes of ascorbic acid stimulation [26]. However, this trend was contradicted by Habermann et al. as their findings suggest a significant increase in ADC within seconds of stimulation with lemon juice (42). Such discrepancies could be explained by the differences in the type and amount of stimulation. However, these studies indicate that DWI is a useful surrogate measure of salivary flow as ADC changes represent the direction of free water, whether that be saliva release from the ducts or reproduction of saliva within the glands. The magnitude of these ADC changes can reflect gland function, with erroneous values indicating presence of disease/damage. Furthermore, by combining the quantitative data of DWI with the visual information of SGS, the functional data can be correlated with gland anatomy to determine the regions of compromise. This will offer the possibility of visualising the heterogeneity of salivary gland radio-sensitivities between each patient. It may also facilitate the early diagnosis of unrecognised glandular diseases and inform key decisions for operative versus non-operative management [42].

Additionally, the adjustment of b values of DWI can present various information from salivary glands. A prospective study by Thoeny et al. investigated the influence of varying b values on the ADC’s of healthy parotid glands, and demonstrated that the selection of lower b values led to higher ADCs [43]. This is because low b values deploy less weighting on diffusion, thus providing a greater description of perfusion parameters such as salivary and blood flow. Conversely, the use of higher b values resulted in lower parotid gland ADCs [43, 44], which is due to the suppression of perfusion effects and thus giving a closer representation of pure diffusion. Therefore, b values reflect the varying levels of contribution from diffusion/perfusion, and practitioners must be aware of these when utilising DWI as it influences ADC analyses. Concomitantly, if b values are standardised during inter-individual analyses, it may elucidate the functional differences (e.g. saliva production and adipose concentration) that exists between each gland. These utilities can provide a baseline comparison for HNC patients such that personalised treatment can be made for better gland sparing.

### Diffusion-weighted imaging in predicting radiation-induced xerostomia

DWI has recently been used for evaluating post-RT salivary gland function and xerostomia severity. A study by Loimu et al. conducted post-treatment ADC analyses of the parotid glands of HNC patients treated with IMRT, to assess if the signal changes can be correlated with SGS. Results showed that irradiated glands exhibit significantly higher maximum ADC values during stimulation compared to pre-irradiation (p < 0.001). A dose–response relationship was also found where glands that absorbed more doses showed higher pre- to post-treatment ADC changes and equated to less salivary flow rates on SGS [9]. Additionally, a post-RT ADC analyses by Zhang et al. on nasopharyngeal cancer (NPC) patients found that those receiving lower radiation doses to the salivary glands had lower maximum ADC measurements during gustatory stimulation and thus had higher likelihood of functional recovery [45]. Table 1 summarises other DWI studies done on salivary gland function, showing similar ADC pattern changes. These results demonstrate that the combination of DWI and gustatory stimulation can be a prognostic tool for the severity of xerostomia.

**Table 1:** Studies of diffusion-weighted imaging assessing post-treatment salivary gland function

Study and type	Objectives	Participants	Methods	Main outcomes
Loimu et al. [9], prospective	Use of DWI for assessment of post-IMRT salivary gland function at rest and during stimulation Matching ADC changes with salivary gland radiation dose, correlating to salivary gland function on scintigraphy	20 patients with HNSCC Treatment: Bilateral neck IMRT with concurrent cisplatin chemotherapy	MRI scanning at a mean 8 days prior to treatment commencement and mean 6 months after completion Post-treatment: DWI series acquired at rest and with ascorbic acid oral stimulation Salivary scintigraphy scanning at mean 3 days prior to treatment onset and mean 6 months after completion. Calculation of salivary ejection fraction	Significant increase in ADC values post-RT in both parotid and submandibular glands (p < 0.001) Significantly higher maximum ADC after stimulation in post-RT vs pre-RT (p < 0.001) Linear correlation between salivary gland RT dose and pre- to post-RT ADC change
Zhang et al. [45], prospective	Use of DWI to assess post-RT salivary gland function during rest and stimulation Follow-up of radiation-induced xerostomia	23 patients with NPC Treatment: IMRT with concurrent docetaxel, cisplatin and 5-fluorouracil chemotherapy	MRI scanning pre-RT, 1 week and 1 year post-RT completion Post-RT: DWI sequences acquired at rest and with ascorbic acid oral stimulation Clinical xerostomia assessment done at the same time as MRI scanning	Significant increase in 1 week post-RT parotid gland ADC at rest and max ADC at stimulation compared to pre-RT (p < 0.001), followed by decrease at 1 year post-RT but still higher than baseline (p < 0.001) Significant increase in 1 week post-RT submandibular gland ADC at rest compared to pre-RT (p < 0.001). No decrease at 1 year post-RT 4 significant predictors for improvement of xerostomia: dose to parotid glands, submandibular ADC at rest, max parotid ADC at stimulation, and time to max parotid ADC during stimulation at 1 week post-RT
Fan et al. [59], prospective	Use of DWI to assess salivary gland function during post-RT follow-up	31 patients with NPC Treatment: Induction chemotherapy followed by helical tomotherapy RT with concurrent docetaxel or cisplatin	MRI scanning at timepoints 2–3 weeks pre-RT and 1, 3, 6, 9 and 12 months post-RT Salivary flow rate and xerostomia assessment conducted with each MRI scan	Significantly higher ADC for parotid and submandibular glands at all post-RT timepoints compared to pre-RT (p < 0.01) Reduced salivary flow rate post-RT, with gradual improvement over time Linear correlation between mean parotid radiation dose and pre- to post-RT change in ADC Significantly higher parotid ADC 1 month and 3 month post-RT in those with mild and moderate xerostomia than those with severe xerostomia (p < 0.05) Positive correlation between salivary gland ADC values post-RT and xerostomia scores post-RT
Shi et al. [60], prospective	Use of DWI with gustatory stimulation to assess salivary gland function pre- and post-RT Correlation of ADC changes with xerostomia scores	30 patients with NPC Treatment: IMRT with concurrent platin-based chemotherapy	DW-MRI scanning before and after RT, follow-up of 12–30 months post-RT DWI sequences taken at rest and after lemon juice oral stimulation Salivary flow rate measurements at resting and stimulated states	Significant increase in resting and stimulated ADC for all salivary glands post-RT compared to pre-RT (p = 0.000) Significantly lower resting and stimulated salivary flow rate in all salivary glands post-RT compared to pre-RT (p = 0.000) Positive correlation between parotid gland ADC at rest post-RT and xerostomia scores post-RT
Zhang et al. [47], prospective	Use of DWI to evaluate early ADC changes in salivary glands during RT Associate mid-RT ADC changes with post-RT xerostomia	26 patients with NPC Treatment: IMRT	MRI scanning pre-RT and two weeks after RT commencement DWI sequences acquired at rest and during ascorbic acid oral stimulation Xerostomia evaluation 6 months post-RT	Significantly higher resting ADC in salivary glands during RT compared to pre-RT (p < 0.01) Negative correlation between ADC changes from pre- to mid-RT with degree of xerostomia at 6 months post-RT

*DWI* Diffusion Weighted Imaging, *IMRT* Intensity Modulated Radiotherapy, *ADC* Apparent Diffusion Coefficient, *HNSCC* Head and Neck Squamous Cell Carcinoma, *MRI* Magnetic Resonance Imaging, *RT* Radiotherapy, *NPC* Nasopharyngeal Carcinoma

Furthermore, the production of ADC histograms provides additional information not revealed within basic descriptive statistics. The ADC histogram provides sets of parameters that can be compared with each other, which reflect the distribution of ADC values and thus representing tissue heterogeneity within the glands [46]. A prospective study by Zhou et al. conducted ADC histogram analyses for parotid gland volumes of NPC patients during pre- and post-RT periods, discovering key trends indicating gland response [46]. During the early stages, the change rates of parotid mean ADC, minimum ADC, kurtosis and several ADC percentiles correlated with the rate of parotid atrophy. Meanwhile, the early change rates of parotid ADC standard deviation (SD) and maximum ADC were higher in those with grade 2 xerostomia compared to grade 1 (p = 0.014, 0.008) [46]. These results suggest that certain parameters from ADC histograms may provide surrogate measurements of radiation-induced tissue damage and be useful predictive markers of clinical xerostomia. However, current studies have only utilised DWI for selected timepoints mid- and post-RT. There is a need for large prospective trials that conduct these analyses continuously throughout the whole treatment course, with comparison to follow-up scans. This will be crucial in establishing a prognostic model that guides the identification of patients at risk, so that treatment plan adaptations and dosimetric adjustments can be done timely to mitigate severe toxicities.

Another factor that influences the prediction of xerostomia is the onset of salivary gland ADC changes in response to RT. A study by Zhang et al. assessed early ADC changes in salivary glands with late xerostomia and found that the mean ADCs of parotid and submandibular glands were significantly higher than pre-RT values as soon as 2 weeks after RT commencement [47]. Additionally, the changes in ADC increase and ADC increase rate of stimulated parotid glands at two weeks were associated with xerostomia severity at 6 months post-RT [47]. A possible explanation for this is the multiple phases of radiation-induced damage. The rise in ADCs during the early period represents the increased molecular flow of functional cells undergoing apoptosis. This is followed by the later onset of xerostomia which is driven by the chronic inflammatory response [48]. Therefore, earlier and greater ADC changes during and immediately after RT could be an indication of more severe xerostomia later, which further necessitates the need for intra-treatment scanning.

### Advanced diffusion-weighted imaging techniques

Additionally, more sophisticated DWI techniques have been developed to improve on the features of conventional DWI. One example is the Intravoxel Incoherent Motion (IVIM) model, where the function separately quantifies tissue perfusion and diffusion [49]. This potentially allows improved assessment of the pathological features of head and neck tumours because during RT the apoptotic process of cells leads to changes in fluid migration, tissue oxygenation and microvasculature [49, 50]. Hence, the use of IVIM provides three additional measures to ADC values: perfusion fraction (*f*), diffusion coefficient of tissue molecular diffusion (D), and perfusion-related diffusion coefficient (D*) [50]. A prospective trial by Marzi et al. assessed the role of IVIM-DWI in predicting the treatment response of cervical lymph nodes in those with head and neck squamous cell carcinomas (HNSCC), and found a significant difference in mid-treatment D values between those with regional control and regional failure (p < 0.05) [50]. The IVIM parameters in the study also had strong correlations with regional control status [50]. Meanwhile, Xiao et al. evaluated IVIM’s ability to predict the response of those with NPC to IMRT treatment and discovered significantly larger changes in D and D* values in treatment responders compared to non-responders [49]. These two studies suggest that IVIM may provide greater diagnostic accuracy compared to ADC analyses, with the additional information of tissue vascularity and oxygenation indicating the radiosensitivity of HNCs.

Another advanced technique is Diffusion Kurtosis Imaging (DKI), where the model accounts for diffusion restriction that occurs within the complicated microstructures of tissues. This may be more accurate than conventional DWI as the latter assumes that water diffusion obeys standard distribution in vivo [51]. DKI parameters include D, the corrected diffusion coefficient for water distribution. Chen et al. explored the use of DKI pre-neoadjuvant chemotherapy in those with NPC to predict treatment response, comparing its prognostic ability to conventional DWI. They found a significant difference in pre-treatment D values between responders and non-responders, whereas no significant difference was observed with ADCs [51]. Thus, DKI may provide a more sensitive prediction than conventional DWI in the early pre-treatment stages for this patient group.

### Caveats of diffusion-weighted imaging

Despite DWI’s unique strengths and potential to supplement HNC management, there are some drawbacks. Firstly, it is challenging to acquire high-quality diffusion-weighted sequences for the region. Head and neck anatomy is complexed, which is challenging for observers to contour. The region also has several air-tissue interfaces, and since most HNCs are of squamous cell origin, they tend to line the epithelial surface close to the air cavities [52]. Areas with multiple air-tissue transitions are especially susceptible to sequence distortions because it disrupts the magnetic field, translating to voxel shifts in the images [52]. Furthermore, movement artefacts such as voluntary motion, vascular pulsation, respiration, coughing, and swallowing contribute to the distortion and cause transient signal loss due to DWI’s sensitivity to motion [33, 52–54]. These artefacts must be mitigated because signal losses influence the averaged DWI data and overestimate ADC values [54].

Secondly, there are inconsistencies in reported ADC values within this field of research. While multiple studies reported in Driessen et al.’s systematic review were able to differentiate malignant tumours from benign lesions using ADC analyses, there is no established consensus on what the ADC threshold for discrimination is [38]. Several factors could explain for the inconsistencies between studies; varying b values, use of different magnetic field strengths, heterogeneity in tumour histology and differing study designs. Apart from tumour assessment, salivary gland analyses are also made difficult as parotid ADC values can be manipulated by factors ranging from malignancies, RT treatment, inflammatory diseases to physiological variations such as adipose content and dietary factors [43, 54]. This emphasises the importance of standardised b values and other imaging parameters during DWI scanning. Therefore, if ADC is to be implemented in standard practice, large studies with clearly defined protocols should be done to establish definitive ADC values with reproducibility.

It is also important to recognise that DWI lacks features that are provided by other functional imaging modes. Rasmussen et al. compared the volumes of interest (VOI) defined by DWI versus FDG-PET images on an integrated PET/MR scanner. The study found that the VOIs from the two only had partial overlap, as the high cellularity provided by DWI showed weak correlation with the glucose uptake represented by FDG-PET [55]. This suggests that the two imaging modalities are complementary; replacing one with another will lead to loss of information. Thus, rather than adopting one imaging technique as a gold-standard, a multi-modal approach should be used. By combining the accurate spatial information of CT, the excellent soft-tissue contrast and cellular information of DW-MRI, and the metabolic activity from PET [34], clinicians can gain a comprehensive assessment of treatment response; a strength of one modality can overcome the caveats of another. Multi-modal imaging can also be used to characterise the heterogeneity that exists between each HNC case and help guide personalised treatments. Future research should investigate how the visual information of imaging can be integrated with patient characteristics (e.g. functional status, metabolic health, histological and blood biomarkers) to achieve comprehensive patient monitoring.

Finally, while prediction of late xerostomia may be possible with multi-modal imaging, such toxicities cannot be prevented even with early detection because the effects of RT would have already been applied. Therefore, the best clinical measures can only mitigate the severity of xerostomia. Currently, intra-treatment interventions include image-guided adaptive RT [2, 4], acupuncture [56, 57] and physiotherapy. Image-guided adaptive RT is especially promising as the intra-treatment adaptations to tumour volume changes may lead to more efficient dose deliveries that permit tissue sparing, thus minimising toxicities [2, 4]. These are improvements to previous pharmacological options such as sialagogues and saliva substitutes, which only offer transient symptomatic management [58]. Further research is needed to incorporate these functional imaging modalities with clinical interventions, namely image-guided adaptive RT, to reduce radiation-induced tissue damage and associated morbidities.

### Review strengths and limitations

This review has its strengths and limitations. Firstly, included studies only involved patients from a population of interest, i.e. locally advanced head and neck cancers. Another strength is that most included studies provide a stratified breakdown of patient characteristics (e.g. demographic details, cancer staging, treatment regimens), which identified possible confounding factors. However, there are some limitations with the included evidence. We admit that excluding studies of patients with oligometastatic status does leave out a large proportion of the HNC cohort, and as a result may only focus on those with long-term survival for late toxicities, whilst assuming that those with distant metastasis may not survive long enough to have late toxicities of significance. Additionally, each study was conducted using different methods, based on protocols and treatment guidelines of their respective era/region. And while many of these studies suggest similar trends, there has been considerable inconsistencies in the quantitative values reported. Such differences make it challenging to establish standards that can be applied universally. Furthermore, several studies were done with small sample sizes (< 30) and inadequate follow-up times, affecting the significance of those results. We acknowledge that these studies may have been restricted by patient ineligibilities or resource constraints.

## Conclusion

In HNC chemoradiotherapy, it is unfeasible to avoid xerostomia as the salivary glands are highly radio-sensitive and often mandatorily included in RT treatment volumes. These side effects remain difficult to assess clinically. Thus, recent research has shifted towards optimising imaging techniques for better treatment assessment and prediction of toxicities to allow for early intervention. The DWI function of MR imaging is of interest due to its high-quality tissue contrast and visualisation of cellular structures, as molecular diffusion can be quantified by ADC values. Existing studies suggest that ADC analyses provide crucial information on tumour differentiation, inter-individual tissue heterogeneity and intra-treatment tissue changes for a comprehensive patient assessment. Future research should validate the use of DWI in conjunction with other functional imaging modalities and correlate imaging data with clinical information, such that prognostic models can be developed to accurately predict patients at risk of severe toxicities.

## Data Availability

Data sharing is not applicable to this article as no datasets were generated or analysed during the current study.

## Abbreviations

3DCRT: Three-dimensional conformal radiotherapy
ADC: Apparent diffusion coefficient
AI: Artificial intelligence
ART: Adaptive radiotherapy
CCRT: Concurrent chemoradiotherapy
CT: Computed tomography
DKI: Diffusion kurtosis imaging
DWI: Diffusion-weighted imaging
FDG: Fluorodeoxyglucose
GTV: Gross tumour volume
HNC: Head and neck cancer
HNSCC: Head and neck squamous cell carcinoma
HPV: Human papillomavirus
IMRT: Intensity modulated radiotherapy
ITV: Internal target volume
IVIM: Intravoxel incoherent motion
LRC: Locoregional control
MeSH: Medical subject headings
MRI: Magnetic resonance imaging
NPC: Nasopharyngeal carcinoma
OAR: Organs at risk
OS: Overall survival
PET: Positron emission tomography
PTV: Planning target volume
QoL: Quality-of-life
RT: Radiotherapy
SCC: Squamous cell carcinoma
SD: Standard deviation
SGS: Salivary gland scintigraphy
VOI: Volumes of interest
